# Sudden diffuse spasm of multiple coronary arteries: A case report

**DOI:** 10.1097/MD.0000000000036889

**Published:** 2024-01-12

**Authors:** Hui Cai, Shuxia Chen, Dongxiao Wang

**Affiliations:** a Department of Cardiology, Hebei General Hospital, Hebei North University, Shijiazhuang, Hebei Province, China; b Department of Cardiology, Hebei General Hospital, Shijiazhuang, Hebei Province, China.

**Keywords:** cardiogenic shock, multi-vessel coronary artery spasms, Valsalva maneuver, variant angina

## Abstract

**Rationale:**

Diffuse multivessel coronary artery spasm (DMV-CAS) was defined as a severe and reversible diffuse spasm occurring in more than 2 major coronary arteries, which is rare in clinical practice. Due to a wide lesion scope, DMV-CAS often occurs in the form of complications. It is not easy to be clinically diagnosed because it is too brief to be caught. Here, we report a rare case of spontaneous subtotal occlusion of 3 major coronary arteries induced by Vasalva action, which was confirmed in real-time by CAG.

**Patient concerns:**

A 68-year-old man had sudden chest pain after forced defecation during hospitalization. The electrocardiogram showed transient ST segment elevation of the inferior wall lead, inversion of the anterior wall, and lateral wall leads T waves. Emergency CAG revealed elongated vessel beds in 3 coronary arteries and multiple diffuse stenosis, but none of the coronary arteries were completely occlusive.

**Diagnoses:**

Diagnoses of DMV-CAS were made based on CAG findings and postmedication response.

**Interventions:**

Nitroglycerin was administered in the coronary arteries. The anti-vasospasm, antiplatelet aggregation and lipid-regulating drugs were administered orally.

**Outcomes:**

The patient was discharged on the 7th day with complete resolution of symptoms and normalization of the electrocardiography findings. No ischemic events occurred during a follow-up for 5 months.

**Lessons:**

This case highlights the identification of multivessel diffuse coronary spasm and acute myocardial infarction, and the prevention of CAS triggers, which requires the attention of clinicians.

## 1. Introduction

Vascular spastic angina (VSA), once known as Prinzmetal angina or variant angina, is a clinical disorder characterized by coronary artery spasms (CAS) that causes resting angina and a rapid response to fast-acting nitrates.^[1]^ However, diffuse and persistent CAS can cause myocardial infarction with electrocardiography (ECG) and myocardial enzyme changes.^[2]^ In most cases, a single epicardial coronary artery is involved; however, coronary artery spasm sometimes occurs in more than one artery and may last longer than in usual angina.^[3]^ Concurrent total ischemia due to multiple coronary artery spasms with clinical manifestations of cardiogenic shock has rarely been reported. Here, we present a patient with transient cardiogenic shock induced by diffuse multivessel coronary artery spasm (DMV-CAS) during hospitalization. Spontaneous subtotal occlusion of 3 coronary arteries in a case confirmed in real time by coronary angiography (CAG).

## 2. Case report

**Figure 1.**
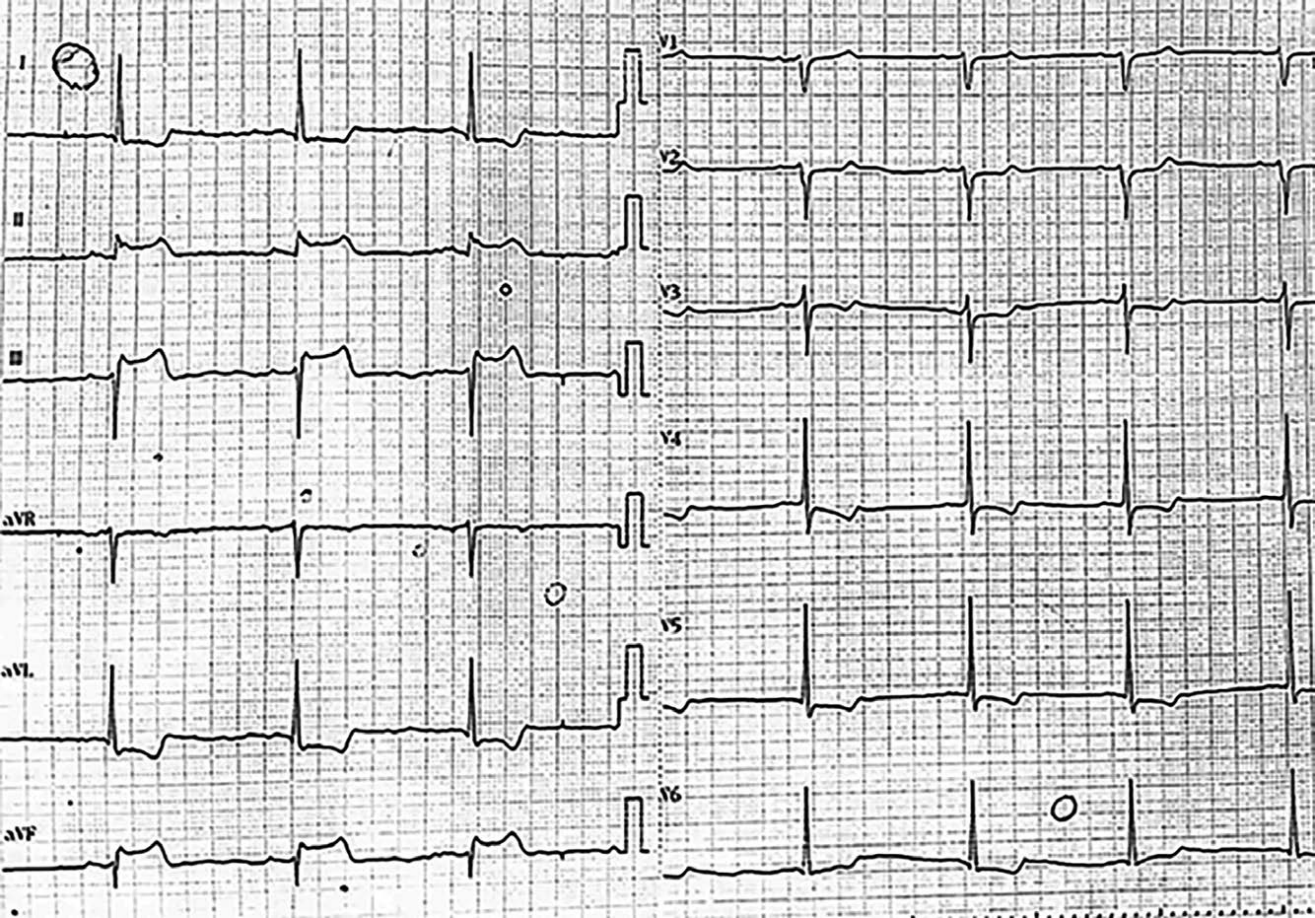
At the onset, the electrocardiogram showed transient ST segment elevation of the inferior wall lead, inversion of the anterior wall, and lateral wall leads T waves.

**Figure 2.**
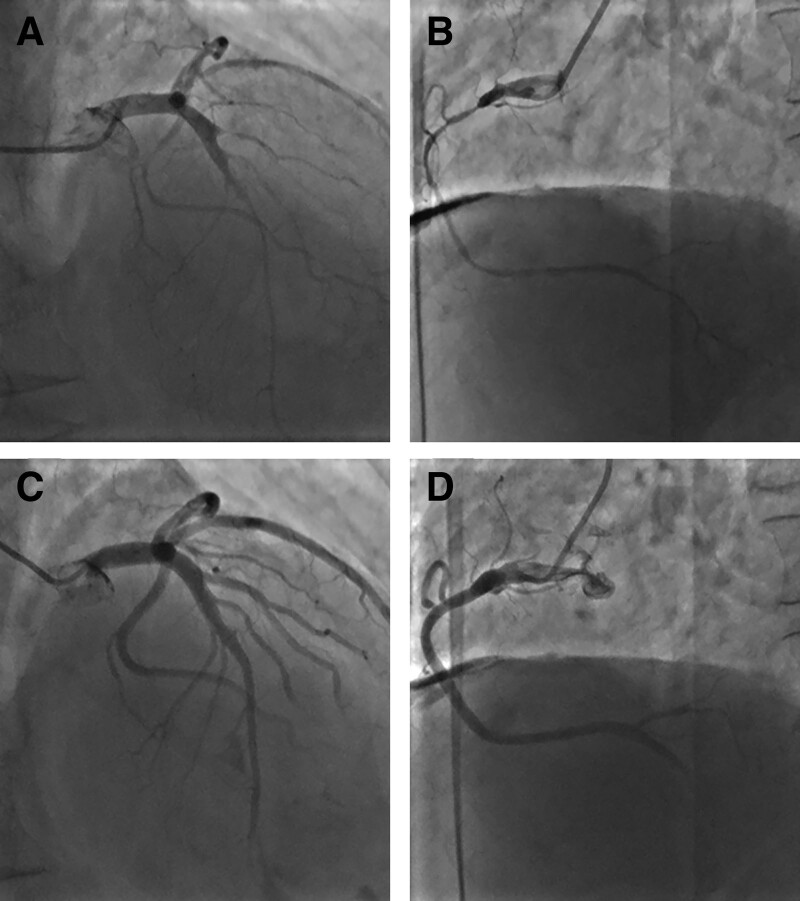
The emergency coronary angiogram demonstrated diffuse spasms of multiple coronary arteries. Panel (A): The left coronary artery. Panel (B): The right coronary artery. A coronary artery that restored normal blood flow after nitroglycerin administration. Panel (C): The left coronary artery. Panel (D): The right coronary artery.

**Figure 3.**
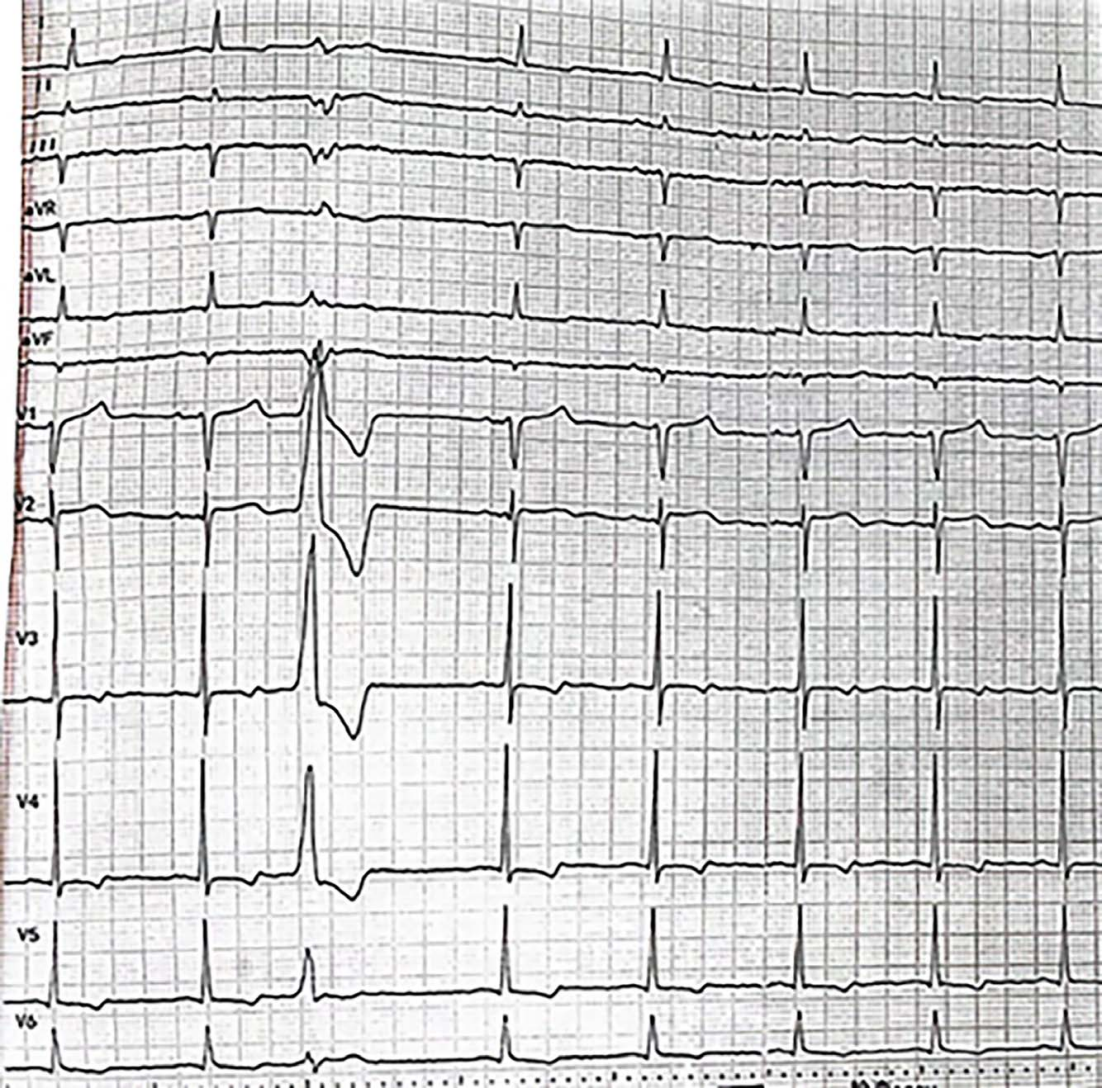
Postoperative electrocardiogram showed an inverted T wave of the anterior wall lead and premature ventricular beats.

A 68-year-old male patient was admitted to the cardiology department with “intermittent dyspnea for more than 4 years and aggravation for more than 3 months.” After admission, the patient was diagnosed with heart failure due to hypertensive heart disease. Routine treatment was administered to correct heart failure, such as strengthening cardiac contractility and diuresis and improving ventricular remodeling. In the early morning of the third day of hospitalization, the patient experienced sudden chest pain and tightness accompanied by sweating and nausea after getting up to force defecation. Blood pressure was 79/64 mm Hg, and the heart rate was 38 beats/min. An ECG on admission revealed sinus bradycardia (HR 46 beats/min) with ST-segment elevation in the inferior leads (II, III, aVF) and ST-segment depression in the lateral and anterior leads (I, aVL, and V2-V6) accompanied by T-wave inversion in leads V2–V6, I, and aVL (Fig. 1). Since he might have an ST-elevation myocardial infarction, immediate treatment was given with dopamine, aspirin 300 mg, and clopidogrel sulfate 300 mg, and emergency CAG was performed. CAG results showed elongated vessel beds and multiple diffuse stenoses in the 3 coronary arteries, but the coronary arteries were not completely occlusive (Fig. 2A and B). After nitroglycerin 400 μg was given to the coronary artery, reexamination angiography showed a significant reduction of stenosis, complete improvement of blood flow in coronary arteries, and enlarged vessel diameter (Fig. 2C and D). The patient’s vital signs were stable during surgery. The postoperative ECG review indicated that the ST segment deviation returned to normal, and sinus rhythm was restored (Fig. 3). The patient reported significant relief of chest discomfort. The usual doses of isosorbide mononitrate, diltiazem, clopidogrel, atorvastatin, and other drugs were subsequently administered. The drugs were administered for a long period after discharge. The patient said that chest pain and tightness did not occur during the 5-month follow-up. This patient had a history of hypertension and had been taking nifedipine controlled-release tablets irregularly for a long time to control blood pressure. Besides, he had a history of gastric ulcer, as well as a history of smoking and drinking. The patient denied drug abuse, excessive emotional stress before and after the onset of the disease, or other related medical histories.

## 3. Discussion

CAS refers to the sudden and strong contraction of the coronary arteries, resulting in complete or subtotal occlusion of the coronary arteries. This causes a sharp decrease (>90%) in the blood flow of the myocardium in the corresponding coronary artery supply area, resulting in transmural myocardial ischemia.^[4]^ CAS can be divided into focal and diffuse types based on the scope of spasm. Focal CAS is more common in the vessels with early atherosclerotic lesions.^[5]^ DMV-CAS causes serious complications such as cardiogenic shock, malignant arrhythmias, and even death, and is more common in patients without obvious coronary artery stenosis.^[6,7]^

Common risk factors for CAS include smoking,^[8]^ Valsalva maneuver, hyperventilation, mental stress, alcohol consumption, and the use of pharmacological molecules, such as sympathomimetic agents, parasympathomimetic agents, and ergot alkaloids.^[9]^ Hypertension, age, and hyperlipidemia are also closely associated with the development of CAS.^[10]^ This patient was a 68-year-old male with a long history of smoking, alcohol consumption, and hypertension. The onset of this case was equivalent to completion of the Valsalva maneuver after forced defecation. Sudden death caused by cardiovascular events in forced defecation is not uncommon, but DMV-CAS has rarely been reported in the literature.

The main pathogenesis of CAS is endothelial dysfunction, autonomic nervous system dysfunction, increased inflammatory response, high reactivity of smooth muscle cells, oxidative stress, and genetic and magnesium ion abnormalities.^[11]^ In this case, the underlying mechanism of CAS may involve the following aspects. First, the patient’s risk factors for smoking, hypertension, advanced age, and alcohol consumption leading to chronic coronary artery inflammation, vascular hyperreactivity, and vascular endothelial dysfunction, which increase the risk of CAS. In addition, the Valsalva maneuver, as a vagus nerve action, can stimulate the vagus nerve, activate the parasympathetic nervous system, and induce autonomic nervous system dysfunction. Vagus predominance is an important mechanism in CAS.^[12]^

The most reliable method for diagnosing CAS is the acetylcholine provocation test, which was previously reported as safe. This test is rarely performed clinically because of serious complications. This can be determined by using captured CAG.^[13]^ Even if the CAG shows that coronary arteries are normal, it does not mean that the coronary artery is not diseased. Intravascular ultrasound can identify the volume and load of plates at the site of small lesions, diffuse coronary intima thickening, and different plaque components, providing additional evidence for the pathogenesis. However, considering the reluctance of patients and their families to further improve intravascular ultrasound, we are unable to determine other possible pathogenesis.

At present, the treatment of VSA is mainly divided into 2 aspects. First, in terms of lifestyle, smoking cessation, control of blood pressure, and blood lipids are extremely important. At the same time, such patients in daily life as far as possible avoid excessive emotional tension, mental pressure, and stimulation of the vagus nerve action. Second, in terms of drug therapy, calcium channel blockers (CCB) are still used as first-line drugs for VSA treatment. CCB combined with long-acting nitrate drugs can produce a cumulative effect in the early stages of CAS, reducing the incidence of CAS.^[14]^ The combination of statins and CCB helps reduce the incidence of coronary vasospasm, especially in patients with high low-density lipoprotein cholesterol.^[15]^ Additionally, Rho kinase inhibitors such as fasudil are effective in preventing acetylcholine-induced coronary spasm and myocardial ischemia in patients.^[16]^ Alpha_1_-adrenergic receptor antagonists may be used for the treatment of antispastic angina pectoris, especially when the combination of DHP CCB and long-acting nitrate does not improve the symptoms.^[17]^ The use of large doses of antiplatelet aggregation drugs may aggravate CAS, which has been reported in the literature.^[18]^ Therefore, we could worsen the patient’s condition by medicating him for ST-elevation myocardial infarction. Intracoronary stenting may be considered if symptoms fail to improve after a combination of these drugs.^[19]^

## 4. Conclusion

CAS is a multi-factorial disease, which is divided into localized and diffuse. It often occurs in the form of complications, so that it is easy to misjudge and misdiagnose. CAS caused by the Valsalva maneuver is rare. Diffuse stenosis involving the 3 coronary arteries is infrequent. Patients have risk factors and predisposing factors for CAS, and the possibility of CAS should not be forgotten when the appearance of myocardial infarction is similar. There is no doubt that there is long-term and regular drug treatment. Lifestyle interventions and vagus nerve stimulation should be avoided. Regular follow-up and observation are necessary.

## Author contributions

**Data curation:** Dongxiao Wang.

**Investigation:** Hui Cai, Shuxia Chen, Dongxiao Wang.

**Methodology:** Hui Cai, Shuxia Chen, Dongxiao Wang.

**Writing – original draft:** Hui Cai.

**Writing – review & editing:** Shuxia Chen.
